# Discovering heritable modes of MEG spectral power

**DOI:** 10.1002/hbm.24454

**Published:** 2019-01-01

**Authors:** Eemeli Leppäaho, Hanna Renvall, Elina Salmela, Juha Kere, Riitta Salmelin, Samuel Kaski

**Affiliations:** ^1^ Department of Computer Science Helsinki Institute for Information Technology HIIT, Aalto University Helsinki Finland; ^2^ Department of Neuroscience and Biomedical Engineering Aalto University Helsinki Finland; ^3^ Aalto NeuroImaging Aalto University Helsinki Finland; ^4^ Department of Biosciences University of Helsinki Helsinki Finland; ^5^ Molecular Neurology Research Program University of Helsinki, Folkhälsan Institute of Genetics Helsinki Finland; ^6^ Department of Biosciences and Nutrition Karolinska Institutet Huddinge Sweden; ^7^ School of Basic and Medical Biosciences King's College London, Guy's Hospital London United Kingdom

**Keywords:** Bayesian reduced‐rank regression, genome‐wide association, GWAS, heritability, magnetoencephalography

## Abstract

Brain structure and many brain functions are known to be genetically controlled, but direct links between neuroimaging measures and their underlying cellular‐level determinants remain largely undiscovered. Here, we adopt a novel computational method for examining potential similarities in high‐dimensional brain imaging data between siblings. We examine oscillatory brain activity measured with magnetoencephalography (MEG) in 201 healthy siblings and apply Bayesian reduced‐rank regression to extract a low‐dimensional representation of familial features in the participants' spectral power structure. Our results show that the structure of the overall spectral power at 1–90 Hz is a highly conspicuous feature that not only relates siblings to each other but also has very high consistency within participants' own data, irrespective of the exact experimental state of the participant. The analysis is extended by seeking genetic associations for low‐dimensional descriptions of the oscillatory brain activity. The observed variability in the MEG spectral power structure was associated with *SDK1* (sidekick cell adhesion molecule 1) and suggestively with several other genes that function, for example, in brain development. The current results highlight the potential of sophisticated computational methods in combining molecular and neuroimaging levels for exploring brain functions, even for high‐dimensional data limited to a few hundred participants.

## 1 INTRODUCTION

Noninvasive brain imaging can, at its best, provide very detailed measures of brain anatomy and function, and of connectivity between different brain areas, but yields very little information on the cellular‐level functions behind the measured phenomena. If variation of neuroimaging features could be associated with genetic variability, it would offer a link to their molecular‐level descriptions and promote better understanding of the significance of neuroimaging measures in brain development, functioning and, eventually, in neurological pathologies.

The search for genetic associations of high‐dimensional brain imaging features is challenging especially due to the typically small experimental group sizes, resulting in weak statistical power (Hibar, Kohannim, Stein, Chiang, & Thompson, 2011). So far, genetic connections to brain imaging have mainly been sought by associating single‐nucleotide polymorphisms (SNPs) of predefined candidate genes with neuroimaging measurements, especially in clinical populations (Egan et al., 2001; Meyer‐Lindenberg, 2010) but increasingly also in healthy participants (Darki et al., 2017; Mueller, Makeig, Stemmler, Hennig, & Wacker, 2011; Smolka et al., 2005). Recently, unrestricted genome‐wide linkage and association analyses have successfully been applied to neuroimaging phenotypes, but so far mainly to fairly simple and prevalent imaging measures, such as the different cortical rhythms (Malone et al., 2014; Porjesz et al., 2002; Salmela et al., 2016; Smit et al., 2017), and auditory evoked responses (Renvall et al., 2012).

An especially prominent feature among human cortical functions is the brain's background activity that covers a wide range of frequencies, including delta (1 − 4 Hz), alpha (≈10 Hz), beta (≈20 Hz), and gamma (≈30 − 200 Hz) bands, featuring both salient rhythmicity and more arrhythmic patterns. The spectral power at delta, alpha, and beta bands has been shown to be highly heritable (Hodgkinson et al., 2010; Salmela et al., 2016; Smit, Posthuma, Boomsma, & Geus, 2005; Smit, Wright, Hansell, Geffen, & Martin, 2006; Van Baal, De Geus, & Boomsma, 1996; Van Beijsterveldt, Molenaar, De Geus, & Boomsma, 1996; Vogel, 1970; Young, Lader, & Fenton, 1972), but still relatively little is known about the underlying genome‐level correlates. The most salient of these intrinsic oscillations are the parieto‐occipital 10 Hz alpha rhythm and the rolandic somatomotor mu rhythm with distinct 10 and 20 Hz components (for a review, see, e.g., Hari & Salmelin, 1997). Both of these rhythms are strongly dependent on the participant's state: for example, the alpha rhythm is attenuated by opening of the eyes, it is modulated by tasks that require visual attention and working memory (Jensen, Gelfand, Kounios, & Lisman, 2002; Tuladhar et al., 2007), and the somatomotor mu rhythm reacts to movement execution and observation (e.g., Hari et al., 1998; Salmelin & Hari, 1994). Both of the rhythms thus appear to have important functional roles instead of only reflecting cortical idling (for a review, see, e.g., da Silva, 2013).

In the present study, we aim at utilizing cutting‐edge computational tools for finding both maximally familial and heritable features from high‐dimensional brain imaging data. We study the wide‐band cortical power spectral structure measured with magnetoencephalography (MEG) in siblings, and establish its potential genetic correlates. We particularly seek to find basic features of the MEG spectral power that would not depend on the participants' exact state. We thus included in the analysis MEG signals recorded in different experimental conditions known to produce variability at the prominent frequency bands (eyes closed, eyes open, simple hand movements). To account for the high dimensionality of both neuroimaging and genetic data, we apply a new Bayesian reduced‐rank regression (BRRR)‐based association study method (Gillberg et al., 2016). Reduced‐rank regression methods have been shown to achieve high power in genome‐wide association studies (GWASs) even when the phenotype dimensionality exceeds the number of participants (Le Floch et al., 2012; Vounou et al., 2012; Vounou, Nichols, & Montana, 2010). We determine a low‐dimensional representation of the MEG spectral power structure that is maximally informative about the relations between the participants. BRRR is subsequently applied for searching for genome‐wide associations of the high‐dimensional MEG spectral power structure, from which it is able to extract heritable components. This demonstrates that association studies for high‐dimensional phenotype can be enabled by extracting lower‐dimensional descriptions of the phenotype in a data‐driven manner. This approach complements association studies of well‐known phenotypes that are derived from the raw data, such as the alpha rhythm.

## 2 METHODS

### 2.1 Participants

Altogether, 210 Finnish‐speaking adults, siblings from 100 families, participated in the study (eight families with three siblings, one family with four). The participants were 30 ± 1 (*SEM*) years old (148 females and 62 males). Monozygotic twins were excluded from the study. Then, 206 participants were right‐handed, three ambidextrous, and one left‐handed. None of the participants had a history of neurological or psychiatric disorders. All participants gave their written informed consent, and the study had a prior approval from the Ethics Committee of the Hospital District of Helsinki and Uusimaa.

### 2.2 MEG recordings

Spontaneous cortical activity was recorded while the participant was seated in the magnetically shielded room of the Aalto NeuroImaging MEG Core, with the head covered by the helmet‐shaped 306‐channel Vectorview neuromagnetometer (Elekta Oy, Helsinki, Finland) that contains 204 gradiometers and 102 magnetometers. Four head‐position‐indicator coils were attached to the scalp, and their positions were measured with respect to three anatomical landmarks (nasion and two preauricular reference points) using a three‐dimensional digitizer, and to the sensor array by briefly feeding current to the marker coils. The measurement consisted of three experimental conditions, with 3 min of data collected for each: (a) eyes closed (hands relaxed), (b) eyes open (hands relaxed), and (c) eyes open and clenching of hands ≈ once per second. Furthermore, to estimate the stability of the recorded brain activities, the study was replicated twice for two participants, with several months between the measurements.

The MEG signals were band‐pass filtered to 0.03–200 Hz and sampled at 600 Hz. For external artifact suppression, a signal space separation method (Taulu & Kajola, 2005) was applied, and each individual MEG recording was transferred to the same head position using a signal space separation‐based head transformation algorithm (Taulu, Kajola, & Simola, 2004), implemented in MaxFilter software (Elekta Oy).

The data analysis was performed on the 204 gradiometer signals. The power spectra in all experimental conditions were estimated using a periodogram of the same length as the input data within MATLAB‐function bandpower, applying a Hamming window. The power spectra were estimated starting from 1 to 3 Hz and widened linearly up to 81.8 − 87.8 Hz, resulting in altogether 21 frequency bands. The band containing 50 Hz was omitted to remove power‐line interference. One sibling pair was left out of the analysis due to noisy data of one sibling.

### 2.3 Genotyping

Autosomal genotypes of the studied individuals were obtained as described earlier by Renvall et al. (2012) and Salmela et al. (2016). In short, genomic DNA extracted from blood samples was genotyped on Affymetrix 250K *StyI* SNP arrays (Affymetrix, Santa Clara, CA) according to manufacturer's instructions. The genotypes were then filtered in Plink (version 1.07; Purcell et al., 2007) based on quality measures of genotyping success (>98% per marker and >95% per individual), minor allele frequency (>5%), and Hardy–Weinberg equilibrium (*p* > .0001 in either of two subsets of 98 unrelated samples). Pairwise relatedness of individuals was checked based on allele sharing, indicating no obviously deflated or inflated relatedness. Individuals whose siblings failed to pass the quality controls were removed as well. The final number of autosomal SNPs was 150,217, while the overall genotyping success rate was 99.8% and the lowest success rate per individual was 97.9%. In the end, high‐quality genotype and MEG data were available for 201 participants, coming from 97 families.

### 2.4 Data analysis

In the following, the MEG data are presented in matrix **Y** with *N* = 201 rows (participants) and *P* = 4,284 columns (204 MEG gradiometers × 21 frequency bands), and the family identifiers in matrix **F** with *N* rows and *M* = 97 columns. The *m*th column of **F**, denoted by **f**_:,*m*_, is a binary vector consisting of 1s for members of family *m* and 0s for the other participants. The genotype is represented as a matrix **G** with *N* rows and *D* = 150,217 SNPs. Each genotype matrix column **g**_:,*s*_ indicates the number of minor alleles for each participant in the specific SNP.

We are interested in analyzing both the familial characteristics of the MEG power spectrum, that is the distribution *p*(**Y**| **F**), as well as the heritable effects *p*(**Y**| **G**). As both *P* and *D* are at least an order of magnitude larger than *N*, the problem needs to be regularized in order to maintain statistical strength. With the knowledge that the variables of **Y** are highly (spatially) correlated, we apply reduced‐rank regression that gives a low‐dimensional projection of the data. Performing a standard regression (or correlation‐based) analysis for a low‐dimensional description of **Y** (such as principal components [PCs]) would be possible as well, but it would not allow taking into account the covariates **F** and **G** in the dimensionality reduction. The study participants were all of Finnish Caucasian origin and their genotyping data were extensively quality‐controlled for in a previous study (Renvall et al., 2012). Population admixture or marker biases were excluded. In the following, the analysis steps are described in more detail.

### 2.5 Bayesian reduced‐rank regression

We first aim at revealing which parts of the MEG power spectrum are similar between siblings. We utilize here BRRR (Gillberg et al., 2016), which simultaneously can predict **Y** given **F**, as well as learn a description of the latent (familial) features of **Y**. The BRRR model is defined as

(1) $$Y=\left(\mathrm{F\Psi}+\Omega \right)\Gamma +E,$$

where **Ψ**_*M* × *K*_ is a low‐dimensional regression coefficient matrix containing the familial values of the latent features, **Γ**_*K* × *P*_ is a projection of the latent space to the MEG channels and frequency bands (the observational space). Here, *K* is a model parameter, chosen by the user, that determines the complexity of the model, and the product **ΨΓ** is a standard regression coefficient matrix **β** with rank *K*. **Ω**_*N* × *K*_ contains unknown factors representing noise in the latent (*K*‐dimensional) space, and **E**_*N* × *P*_ describes residual noise in the observation space—in this case, also the differences between siblings. Specifying the noise models in this way is useful particularly when noise is correlated with signal (as in this case across the channels), as demonstrated by Gillberg et al. (2016). Any categorical and numeric variables, such as gender and age, could be included in the covariate matrix in Equation (1), but here we use only the family identifier matrix **F** to focus on inferring familial features.

The regression and projection coefficients are given shrinkage priors presented by Gillberg et al. (2016); these aid in solving the identifiability problem inherent in reduced‐rank regression by placing the strongest effects in the first components. Specifically, each column **ψ**_:*k*_ and row ***γ***_*k*,:_ is given the prior $N\left(0,\tau_{k}^{-1}I\right)$, where $\tau_{k}=\prod_{l=1}^{k}\delta_{l}$, with $\delta_{1}∼G$(10,1) and $\delta_{l>1}∼G$(4.1,1), with N denoting the normal distribution and G denoting the Gamma distribution, parameterized by shape and rate. As the Gamma distributions have positive expected values, the precisions *τ*_*k*_ increase with *k* and limit later components to vary less than the earlier ones. Furthermore, a small fixed *K* is used to control the model complexity, and hence only low noise levels are assumed: elements of **E** and **Ω** are set to have mean 0 and *SD* 0.1 and $10^{-6}\tau_{k}^{-1}$, respectively. For model inference, we initialize the **Γ** and **Ψ** such that the family identifiers explain maximal amount of variance in **Y**, that is, according to linear discriminant analysis, each component explaining less variance than the previous one. This kind of informed initialization allows the sampling to converge rapidly. The parameters are inferred using Gibbs sampling with 500 iterations, discarding the first 250 as a burn‐in period. The proportion of total variance explained by the covariates approximately converged within the burn‐in period of 250 Gibbs samples.

**Figure 1:**
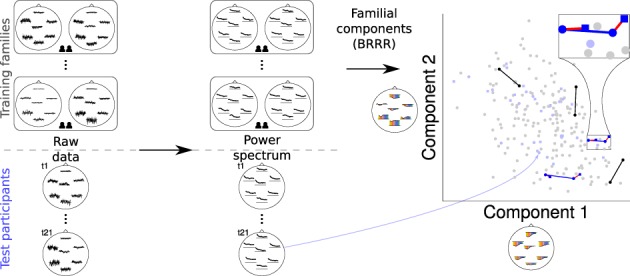
Cross‐validation procedure for estimating the quality of the identified familial structure. Left: Raw data of training families (illustrated here for 1 s on seven magnetoencephalography [MEG] channels) is used for computing spectral power, which in turn is used to estimate familial components (here components 1 and 2) based on data of siblings. Right: Each participant is visualized as a point in the here two‐dimensional familial component space. Three families included in the training data (black), as well as two test families (blue) are highlighted by connecting lines between the siblings. Furthermore, components of test participants based on two time periods in the data (connected by red line; see insert at top right) demonstrate the robustness of the structure within individuals [Color figure can be viewed at http://wileyonlinelibrary.com]

As only the family identifier matrix **F** is used as a covariate in Equation (1), the model cannot differentiate siblings within families, and only aims to maximally explain differences between the families. This leads to the rows of **Γ** directly revealing features that are maximally different between families, as well as maximally similar within families. To evaluate the ability of BRRR to discriminate between families, we perform cross‐validation such that **Γ** is learned from training data (90% of the families) and the similarity of the test participants is estimated in the latent space **YΓ**^−1^. This process is illustrated in Figure 1. If the model is successful in extracting heritable components, siblings should be located nearby in the latent space (as measured by L1 distance). We examine the performance separately in the three experimental conditions, and by using MEG data fragments of different lengths. We also test how consistently **Γ** identifies the same participant from earlier versus later parts of the same experimental condition, as well as across different conditions, that is, whether the latent components can be considered as a “cortical fingerprint.” Campisi and Rocca (2014) reviewed the use of this kind of fingerprints in Electroencephalography (EEG)‐based user recognition. In addition, we are interested in how large the within‐family differences are compared to the between‐family ones. This is accomplished by inspecting the proportion of total variance explained (PTVE) in **Y** by a rank *K* BRRR solution.

### 2.6 Genome‐wide association study

Finally, BRRR is used to perform a GWAS, similarly to Marttinen et al. (2014), with additional family identifier covariates explaining family‐related environmental effects separately. A separate model is trained for each of the 150,217 SNPs as:

(2) $$Y=\left(\left[F\;g_{:,s}\right]\Psi +\Omega \right)\Gamma +E,$$

where the covariates are the family identifiers **F** and **g**_:,*s*_, the genotype for SNP *s*. The additional covariate is included in **Ψ**, which is now a matrix in ℝ^(*M* + 1) × *K*^, describing the *M* families and the single SNP in the latent space. Matrix **Γ** maps the latent space, both for the familial indicators and the SNP, into the sensor space, as in Equation (1). This approach allows us to jointly study the *P* = 4,284 highly correlated spatiospectral MEG features (in matrix **Y**), while preserving high statistical strength, as the regression in Equation (1) is of low rank (Vounou et al., 2010). Thus, we can effectively search for associations for *K*_*G*_ << *P* components that are estimated using the observed SNP and family information. Here choosing a higher number of components allows searching for associations for a broader range of latent features, whereas a lower number maintains higher statistical strength. The model estimates a set of components that explain joint effects of SNPs and family effects. It is initialized and inferred as described in the previous section; the spectral MEG components are initialized to maximally separate families, but each SNP may affect this structure and create unique outcomes.

We do not explicitly account for population stratification in Equation (2), which could potentially lead to spurious results. All our participants represented the Finnish population, which is known to harbor internal genetic structure, with the main stratification observed between East and West Finland (Kerminen et al., 2017; Salmela et al., 2008). To confirm the absence of significant population stratification effects, we used a population sample of 265 Finns with known grandparental birthplaces across the main genetic clines of Finland (Salmela et al., 2008) to calculate the first 10 PCs of the population data, based on the genotypes of 136,370 SNPs shared between that data set and ours. These PCs were then used to calculate the corresponding scores for all the 201 participants of our study. This was done using the program SMARTPCA as implemented in EIGENSOFT (Patterson, Price, & Reich, 2006; Price et al., 2006). None of the correlations of the first PC, corresponding to the East–West origin, with the six MEG components (i.e., phenotypes) acquired in the familial BRRR analysis were significant (maximal *r* ^2^ = 0.01 with *p* = .16). We further examined the correlations of the other 9 PCs with the MEG components, and found no significant correlations (maximal *r* ^2^ = 0.05 with p = 1.3×10^−3^ between PC9 and MEG component 6, with Bonferroni corrected significance limit at 8.3×10^−4^). Furthermore, PC9 showed negligible correlation with the other components of MEG spectral power, thus explaining only little variance in total. We therefore conclude that any population stratification is highly unlikely to affect the results of our association study.

We determine the associations in this study based on the proportion of total variance explained by the SNPs. Additional convergence and stability checks are performed for the models resulting in significant or suggestive findings. Convergence is checked by running a longer Gibbs sampling chain for 5,000 iterations: these runs resulted in similar parameters and PTVEs as the shorter ones (500 iterations). Model stability was evaluated by running sampling chains from different random initializations. The deviation between PTVEs acquired for the same data with different sampling chains was minimal (maximal difference in PTVE 0.02%), in line with (Gillberg et al., 2016).

We produce a random baseline for the association study through permutation testing. Ideally, hundreds of batches of 150k permuted runs would be used to estimate the significance limits for the PTVE values acquired from the nonpermuted runs. However, inferring the BRRR parameters for a single SNP requires approximately 1 CPU hour, prohibiting multiple repetitions of 150k runs. Thus, we analyze a total of 150k runs where the genotype is permuted, and compare the distribution of the resulting PTVE values to the PTVE values in the original runs (instead of the distribution of the maximal PTVE values repeated over hundreds of batches of 150k runs).

As computational reasons prohibit estimating *p*‐values based on the permutation tests, we assess the significance of the acquired PTVEs by estimating the local false discovery rates (LFDRs) using an empirical Bayes approach (Stephens, 2017). The approach is designed for multiple testing problems, taking into account also the variance of the estimates. The *SDs* of the nonpermuted PTVEs are set as the *SE* for the significance testing. We report both the significant (LFDR <0.05) and suggestive (LFDR <0.1) findings. The resulting PTVEs are visualized using an R script modified from (Saxena et al., 2007), accompanied with recombination rates based on the Finnish sample of the 1000 Genomes data (available at ftp://ftp.1000genomes.ebi.ac.uk/vol1/ftp/technical/working/20130507_omni_recombination_rates/).

## 3 RESULTS

### 3.1 Familial structure

**Figure 2:**
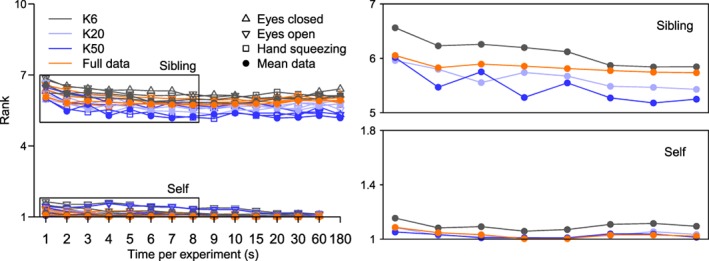
Mean similarity rank of a sibling to a test participant (left, top), and the similarity rank of a test participant to his/her own data at a different time period in the same experimental condition (left, bottom); shown as a function of seconds of data used for evaluation. The similarities are based on 6, 20, or 50 familial components, or the full magnetoencephalography (MEG) spectral power data. Zoom‐ins of both (sibling and self) are shown on the right, for data averaged across the experimental conditions. The test set consists of 1 related (sibling or self at another time period) and 18 unrelated participants: thus, perfect prediction corresponds to rank 1, and a random prediction to rank 10 [Color figure can be viewed at http://wileyonlinelibrary.com]

We first examined the effectiveness of BRRR in identifying components that are similar within families. The examination was performed for time periods of different lengths (from 1 to 180 s), for each in a 10‐fold cross‐validation scheme, where 10% of the families were assigned as test data, and the model was estimated based on the remaining participants, using the family identifiers **F** as covariates. Figure 2 (“Sibling”) illustrates the ranking of siblings as a function of the length of the time period per participant, both separately for the three experimental conditions and as an average across them (denoted as “mean data”). The ranks are shown for different numbers of components *K* = {6, 20, 50}. Monotonic improvement with *K* was observed, with ranks reaching their minima at *K* ≈ 50. All the experimental conditions resulted in very similar accuracies in ranking the siblings. The accuracies of the models in finding any test participant's sibling among a group of 18 unrelated participants improved when more data were included, up to time period length of about 5–7 s. The best average ranks for the siblings were obtained by averaging over experimental conditions, with ranks ranging between 5 and 6 for segments longer than 5 s (1 denoting perfect accuracy and 10 a random guess).

When models with different numbers of components were compared to the baseline (i.e., the full data **Y** used in computing the similarities based on L1 distance), as few as *K* = 6 latent features yielded nearly as good accuracies as using the full 4,284 features of **Y**. Use of larger number of familial components allowed picking out relevant familial structure and leaving out noise, thus outperforming the baseline ranking, which is based on the full data.

For establishing the consistency of the extracted components within participants, we tested the model's ability to identify data recorded from the same participant at another time point. The results (“self” in Figure 2) demonstrate that even a few seconds of data were sufficient for identifying test participant's own data among other participants' data sets. Overall, any participant could be identified with an average rank of ≈1.1 when 60 s of data was used. Furthermore, participants could be identified accurately irrespective of which experimental condition was used as the test data, with the exception that with few seconds of recordings only, “eyes closed” differed significantly from the two other conditions. Combining the different conditions resulted in the smallest rank of ≈1.015 (all but one of the participants identified perfectly in the cross‐validation) when using only 3 s of data from each condition (*K* = 20).

We additionally assessed the identification accuracy in two participants with multiple recording sessions several months apart. With tens of seconds of data, and by averaging over the experimental conditions, the difference between recordings was negligible, suggesting that the low‐dimensional familial components can provide a robust individual fingerprint.

**Figure 3:**
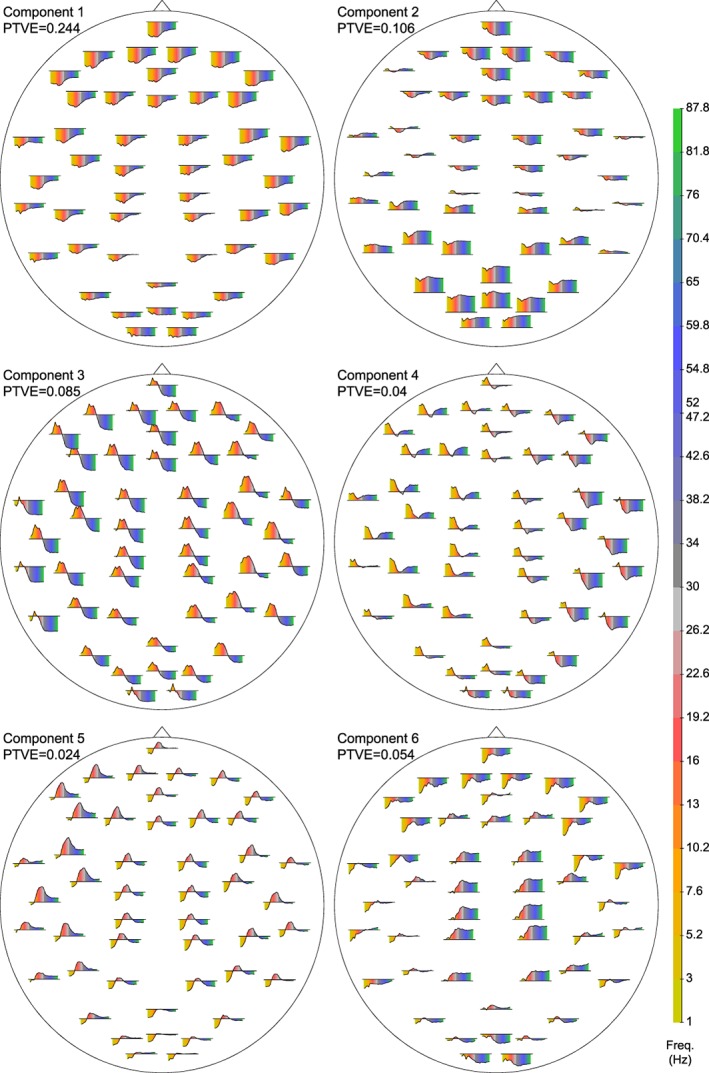
Six BRRR components best explaining the brain activity differences between families. The varying component weights over frequencies (1–88 Hz) on a subset of gradiometer sensors are illustrated on the magnetoencephalography (MEG) sensor plane (all 204 sensors are shown in Supporting Information). For visualization, each frequency bandwidth (in Hz) is given a color shown on the right. The measurement helmet is viewed from above, flattened onto a plane, with the nose pointing upward. The 204 planar gradiometers of the Vectorview system are arranged in 102 locations along the helmet [Color figure can be viewed at http://wileyonlinelibrary.com]

**Figure 4:**
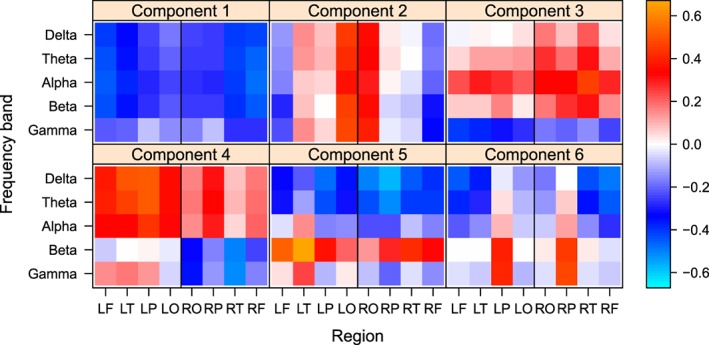
Summary of the weights of the six BRRR components that maximally explained the brain activity differences between families. The component weights (shown in Figure 3) are averaged over distinct frequency bands and channels covering frontal (F), temporal (T), parietal (P), and occipital (O) areas over the left (L) and right (R) hemispheres [Color figure can be viewed at http://wileyonlinelibrary.com]

Combination of the experimental conditions resulted, on average, in the smallest ranks in both sibling and self prediction, and was thus used in the following analysis. To illustrate the spectral data structures found by BRRR, we estimated the model on the full MEG spectral power data. The family identifiers were again used as covariates, with *K* = 6 and *K* = 20. For these familial component numbers, the PTVEs in **Y** were approximately 54 and 61%, respectively. Figure 3 depicts the familial components acquired with *K* = 6. The parameter matrix **Γ** describing the projection of the low‐dimensional variables into **Y** is visualized on the MEG sensor plane where each gradiometer pair has its own weights for the different frequency bands. The observed components are spatially smooth, but a clear structure can be seen with respect to different frequency bands and brain areas. For clarity, only a subset of the gradiometers are illustrated in Figure 3, and the complete figure can be found in Supporting Information. Component 1 incorporated the total power as a major determinant of the overall data variability, with 24.4% of PTVE, extending to frequency bands up to 90 Hz. Component 2 (with 10.6% of PTVE) reflected spatial variation of total power, with increased power at the occipital channels and reduction at the frontal channels. Component 3 (8.5% of PTVE), in turn, highlighted spectral variation of total power, as a combination of increased power up to ≈25 Hz and reduction in the gamma band (here 25–90 Hz). Figure 4 provides a summary of the components clustered on both the MEG channels and frequency bands.

### 3.2 Genome‐wide association study

Finally, we performed a GWAS by inferring independent BRRR models for each of the 150k SNPs, using the family identifiers and one SNP at a time as covariates. Given the relatively small number of participants, we set *K* = 6 to keep the model complexity low. Yet, this number of components sufficed to capture meaningful features of the phenotype. We examined the PTVEs of the SNPs explaining the MEG spectral power measurements.

We found six SNPs significantly associated with oscillatory brain activity (LFDR <0.05) and further eight SNPs suggestively associated (LFDR <0.1). Three significantly associated SNPs are located close to gene *SDK1* on chromosome 7, and one distant from known genes on chromosome 6. Out of the six significant findings, we regarded two as uninteresting in the present context, as they simply indicated the gender of the participants, explaining differences in the total spectral power. The rest of the SNPs along with their closest genes (annotations obtained from USCS Genome browser, assembly GRCh37) are reported in Table 1. We additionally studied possible eQTL effects of the implicated SNPs (examining brain‐tissue specific significant effects in https://gtexportal.org/home/datasets), recognizing, however, that many significant associations cannot be easily explained by eQTLs (GTEx Consortium, 2017). We identified significant eQTL associations for two suggestive SNPs, rs13057362 (gene DRICH1) and rs4622752 (genes LONRF2 and CHST10) in seven and two brain regions, respectively. No significant eQTLs were detected for the other implicated SNPs.

**Table 1:** SNPs resulting in significantly or suggestively high PTVE in the MEG spectral power measurements. Significance was estimated using LFDR <0.05 (denoting significant findings, above the dashed line). The chromosome where the SNP was located and up to two closest genes within 70 kb are reported, with genes known to be expressed in the brain bolded. Two significant SNPs, indicating the gender of the participants, were omitted from the table

PTVE	LFDR	SNP	Chromosome	Closest genes
0.036	0.0097	rs2040918	7	**SDK1**
0.035	0.015	rs6454976	6	
0.032	0.043	rs5021672	7	**SDK1**
0.032	0.043	rs11773381	7	**SDK1**
0.032	0.058	rs747995	6	FRMD1
0.032	0.058	rs747994	6	FRMD1
0.032	0.06	rs13057362	22	CES5AP1,**BCR**
0.031	0.065	rs4622752	2	**AFF3**
0.031	0.066	rs2241220	12	**SSH1**
0.031	0.071	rs16925246	10	**CTNNA3**
0.031	0.082	rs3895695	5	**CTNND2**
0.03	0.087	rs10510836	3	**FHIT**

AFF3 = AF4/FMR2 family member 3; BCR = breakpoint cluster region; CES5AP1 = carboxylesterase 5A pseudogene 1; CTNNA3 = catenin alpha 3; CTNND2 = catenin delta 2; FHIT = fragile histidine triad; *FRMD1 =* FERM domain containing 1; LFDR = local false discovery rate; MEG = magnetoencephalography; PTVE = proportion of total variance explained; SDK1 = sidekick cell adhesion molecule 1; SNP = single‐nucleotide polymorphisms; SSH1 = slingshot protein phosphatase 1.

**Figure 5:**
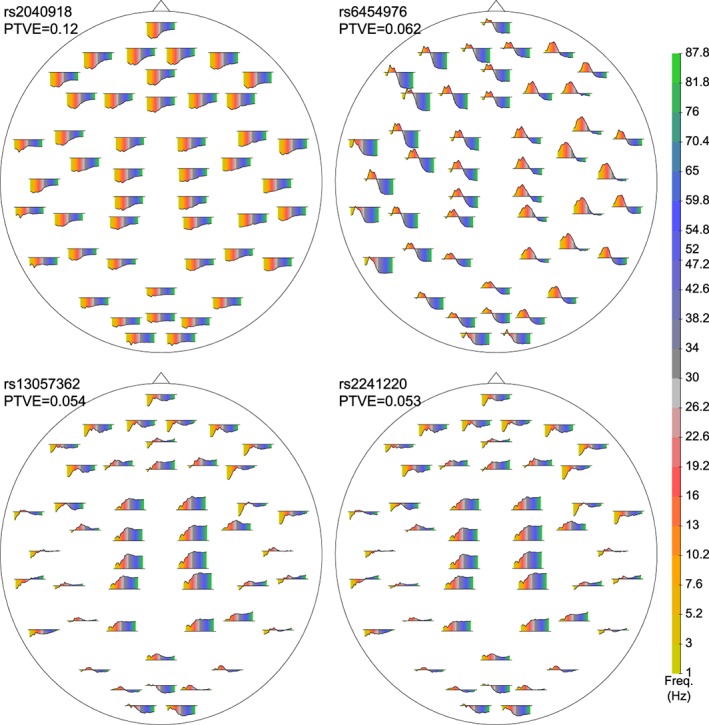
Components significantly explained by individual single‐nucleotide polymorphisms (SNPs), with proportion of total variance explained (PTVE) shown. Each associated SNP explained variance in the total spectral power, similar to rs2040918 (top left) here; SNPs explaining other spectral power structures as well are illustrated. The varying component weights over frequencies (1–88 Hz) on a subset of gradiometers are visualized on the magnetoencephalography (MEG) sensor plane (all 204 sensors are shown in Supporting Information). Each frequency bandwidth (in Hz) is given a color shown on the right [Color figure can be viewed at http://wileyonlinelibrary.com]

**Figure 6:**
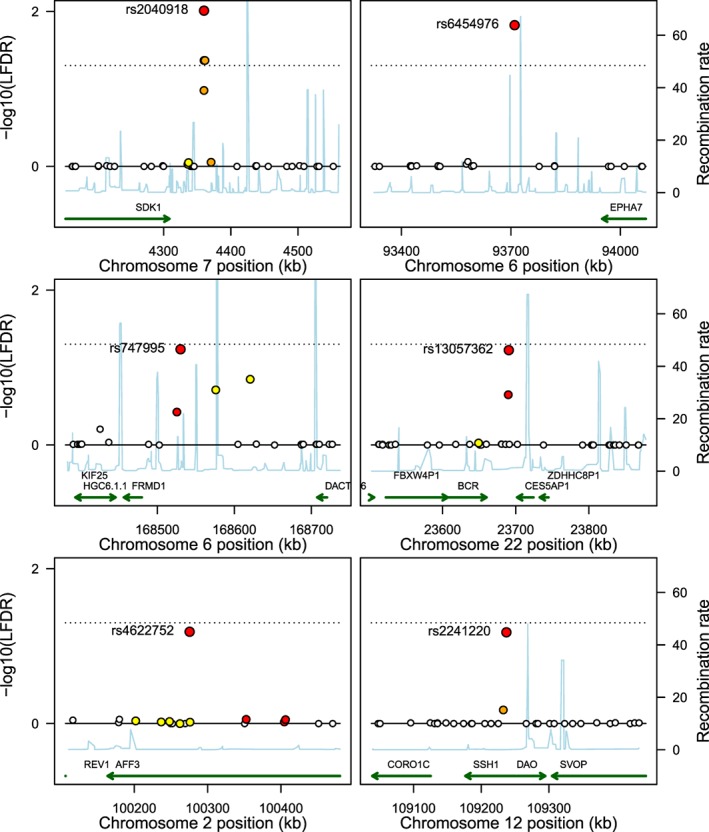
Regional association plots showing the local false discovery rate (LFDR) of single‐nucleotide polymorphisms (SNPs) near the most significant findings, with significance limit 0.05 indicated by the dotted line. Each nearby SNP is colored according to its linkage disequilibrium with the most significant SNP of the region (*r*^2^ > 0.8 red, *r*^2^ > 0.5 orange, and *r*^2^ > 0.2 yellow). The light blue line indicates local recombination rate ($\frac{\mathrm{cM}}{\mathrm{Mb}}$); its peaks are expected to delineate the regions of strong disequilibrium. Genes located in the area are depicted in dark green. All genomic coordinates are derived from human genome assembly GRCh37. Six regions containing the lowest LFDR values are illustrated [Color figure can be viewed at http://wileyonlinelibrary.com]

All the associated SNPs strongly explained the MEG total spectral power (relatively constant weights across the frequency bands and the scalp; top left in Figure 5), which emerged as the most varying component of oscillatory brain activity across participants in this study. Some SNPs explained additionally other spectral power structures, as illustrated in Figure 5 (and for all the gradiometers in Supporting Information). Overall, the resulting heritable components strongly resembled the familial components illustrated in Figure 3. We additionally inspected the PTVEs in the SNPs surrounding the significant associations to address their robustness. Loci that are near to each other in the genome tend to show linkage disequilibrium, that is, their alleles correlate. Therefore, phenotypic associations are typically visible in several markers within the associated region, and the presence of such associations can serve as an additional control of result quality. Figure 6 illustrates these regional association plots. It demonstrates the robustness of the *SDK1* association, as nearby SNPs result in low LFDRs.

**Figure 7:**
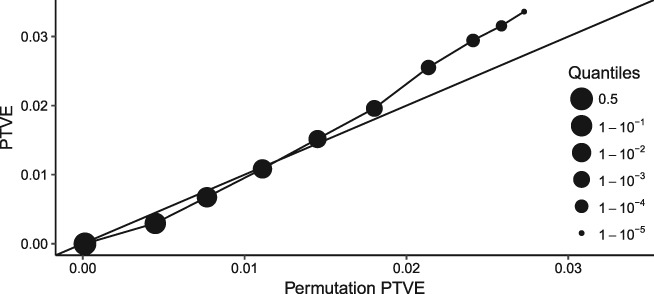
Q–Q plot showing the proportion of total variance explained (PTVE) quantiles of the original Bayesian reduced‐rank regression (BRRR) runs against those of runs with permuted single‐nucleotide polymorphism data

The PTVE quantiles for the heritable components, estimated from the original data, were compared to those resulting from the permuted runs (Figure 7), illustrating that the SNPs achieve better‐than‐random explanation of the data. None of the 150k runs with permuted SNPs resulted in false discovery rates below 0.05.

The presented analysis requires setting a fixed dimension for the latent space, that is, the parameter *K*. Using a suitable sparsity prior would allow for inferring *K* from the data, as shown by Gillberg et al. (2016). However, for the repeated inferences performed here, we wanted to unify and simplify the approach by setting a fixed *K* = 6 for each of the models. To inspect the effect of this choice in the association study results, sensitivity analysis was performed by repeating the study with *K* = 1, …, 30 for 15 SNPs resulting in the highest PTVEs (with *K* = 6), as well as for 100 additional randomly selected SNPs. Robust results were seen across the tested values of *K*: for the top 15 results, maximal deviance from the PTVE obtained with *K* = 6 was 0.005 (0.003 when omitting overly simple *K* = 1, 2). Furthermore, the two highest associations reported in Table 1 would have been significant (LFDR <0.05) with all the tested *K* ≥ 3. The two other significant results with LFDR ≈0.043 (*K* = 6) would have been significant with 12 of the 30 tested component numbers. For the 100 randomly selected SNPs, the maximal PTVE difference from values computed with *K* = 6 was 0.008 (0.005 with *K* ≥ 3).

Genes expressed in the brain and thus with potentially interesting functions relevant for this study included *SDK1* (sidekick cell adhesion molecule 1; Saus et al., 2010; Tsang et al., 2013), *BCR* (breakpoint cluster region (Hashimoto et al., 2005), slingshot protein phosphatase (Xiang et al., 2014), catenin alpha 3 (Morgan et al., 2008; Stahn et al., 2016), catenin delta 2 (Belcaro et al., 2015; Jun et al., 2012), and fragile histidine triad (Corominas et al., 2014). We visualized the expression of these genes in Supporting Information over different brain areas for cell line, fetal, newborn, and adult samples. The expression profiles were extracted from FANTOM5 human promoterome and gene expression data (Andersson et al., 2014) using ZENBU (http://fantom.gsc.riken.jp/zenbu/). The FANTOM5 project has established the transcription start sites for most human genes in a large number of tissue and cell culture samples, yielding a map of most gene promoters (the promoterome) and tissue‐specific gene expression data. In case of multiple samples regarding the same target, we are showing their median. Furthermore, even though FERM domain containing 1 shows no expression in the brain, and is hence not show in the Supporting Information, Ebejer et al. (2013) have indicated a suggestive connection between the gene and ADHD.

## 4 DISCUSSION

We demonstrated in the present study that BRRR can efficiently infer familial structures from brain activity measured by MEG. The identified salient components in MEG power spectra predicted siblings well above chance level, despite the relatively small number of participants. We further discovered that the MEG spatiospectral composition of any individual participant can be reliably identified based on only a few seconds of data, and mostly independently of the experimental conditions or time interval within the measurement, suggesting that it may be considered as representing an individual “cortical fingerprint.” Furthermore, application of BRRR on gene data resulted in MEG spatiospectral components which largely agreed with the components identified on the basis of familial structure alone, with a significant genetic link to *SDK1* in chromosome 7, as well as suggestive links to several other genes expressed in the brain. Previous analysis of this data collection regarding genetic background of the 10‐Hz parieto‐occipital rhythm pointed to chromosome 10 with several plausible genes (Salmela et al., 2016). The *SDK1* gene has been implicated previously in neuronal connectivity in the retina (Yamagata & Sanes, 2008), but a survey of its expression pattern in the human brain in the FANTOM5 database revealed high expression in the human brain, including fetal occipital and parietal lobes, adult hippocampus, substantia nigra, parietal cortex and spinal cord, suggesting wide‐spread functions in neural processing. Thus, the functions and expression pattern of this gene are generally compatible with our current findings.

The discovered latent components were sensible from a neurophysiological point of view. In line with earlier EEG studies (e.g., Smit et al., 2005; Van Beijsterveldt et al., 1996; Young et al., 1972), the overall oscillatory power was shown to be highly heritable and to extend also to higher, gamma‐range (30–90 Hz) frequencies. The overall spectral power was subsequently shown to suggestively associate with loci on multiple chromosomes, containing genes with known relevance for brain function. The significantly associated gene *SDK1* encodes a member of the immunoglobulin superfamily (Yamagata, Weiner, & Sanes, 2002) that mediates laminar connections especially in retina, but likely also in other parts of central nervous system (Yamagata & Sanes, 2008). Copy number variations in *SDK1*, among several other genes, have been associated with schizophrenia in the Asian population (Sakai et al., 2015).

The reduced‐rank regression framework proved to be well suited for a GWAS. Learning a compact set of latent features and searching for the associations jointly, along with performing the analysis separately for each SNP, was shown to be successful here, allowing the inference of maximally heritable components. In contrast, even though, for example, PCA preprocessing could be used to maintain high statistical power in GWAS, it would limit the study to consider the *K* PCs computed from the phenotype only, irrespective of their heritability. Furthermore, explaining the phenotype jointly with the family identifiers and the SNPs allows utilizing all the observations for GWAS. In contrast, approaches such as PC of heritability (Ott & Rabinowitz, 1999), require splitting the data to separately estimate the familial components and the associations (Klei, Luca, Devlin, & Roeder, 2008). Analysis of the statistical power of reduced‐rank regression on high‐dimensional data, simulated to resemble imaging genetics, has been presented in (Vounou et al., 2010).

The performed association study is limited by the small number of participants, and thus further verification of the observed associations is required for drawing strong conclusions. We used a moderate‐density set of SNPs to analyze genetic associations, and therefore additional associations might be detected in further studies using SNP arrays with higher density. The main goal of the study was to demonstrate the prospects of reduced‐rank regression for challenging problems limited by the amount of data, as was the case here. In addition, in order to properly estimate the robustness of the individual MEG spectral power fingerprints, several recording sessions will be needed for a large sample of the participants.

Even though the reduced‐rank regression was shown to provide robust fingerprints of brain activity and enable GWAS in a challenging domain, some aspects remain for future research. Firstly, speeding up the inference would allow (a) easier scaling of the analysis to larger sets of measurements, and (b) computing a larger number of permutation comparisons. Also, taking into account the full genome while searching for associations with a single SNP would allow leveraging statistical power over correlated SNPs, but this approach remains extremely challenging especially with high‐dimensional phenotypes.

In this study, we searched for familial and heritable features of the MEG spectral power during rest conditions and continuous task performance. The same approach may be extended to event‐related experimental designs to shed light on how different stimulus‐evoked processes in the brain are genetically determined. In future studies, extending this type of analysis to the source space will enable further interpretation of associations between neural signals and genes via engagement of specific brain regions.

## Supporting information


**Supporting Information S1**

**Supporting Information S2**

**Supporting Information S3**

**Supporting Information S4**

**Supporting Information S5**
