# Supplementary figures and images for: Discovering heritable modes of MEG spectral power
Source: Hum Brain Mapp. 2019 Jan 1;40(5):1391–402. doi: 10.1002/hbm.24454 (PMC6590382; doi:10.1002/hbm.24454)

rs2040918  
PTVE=0.12

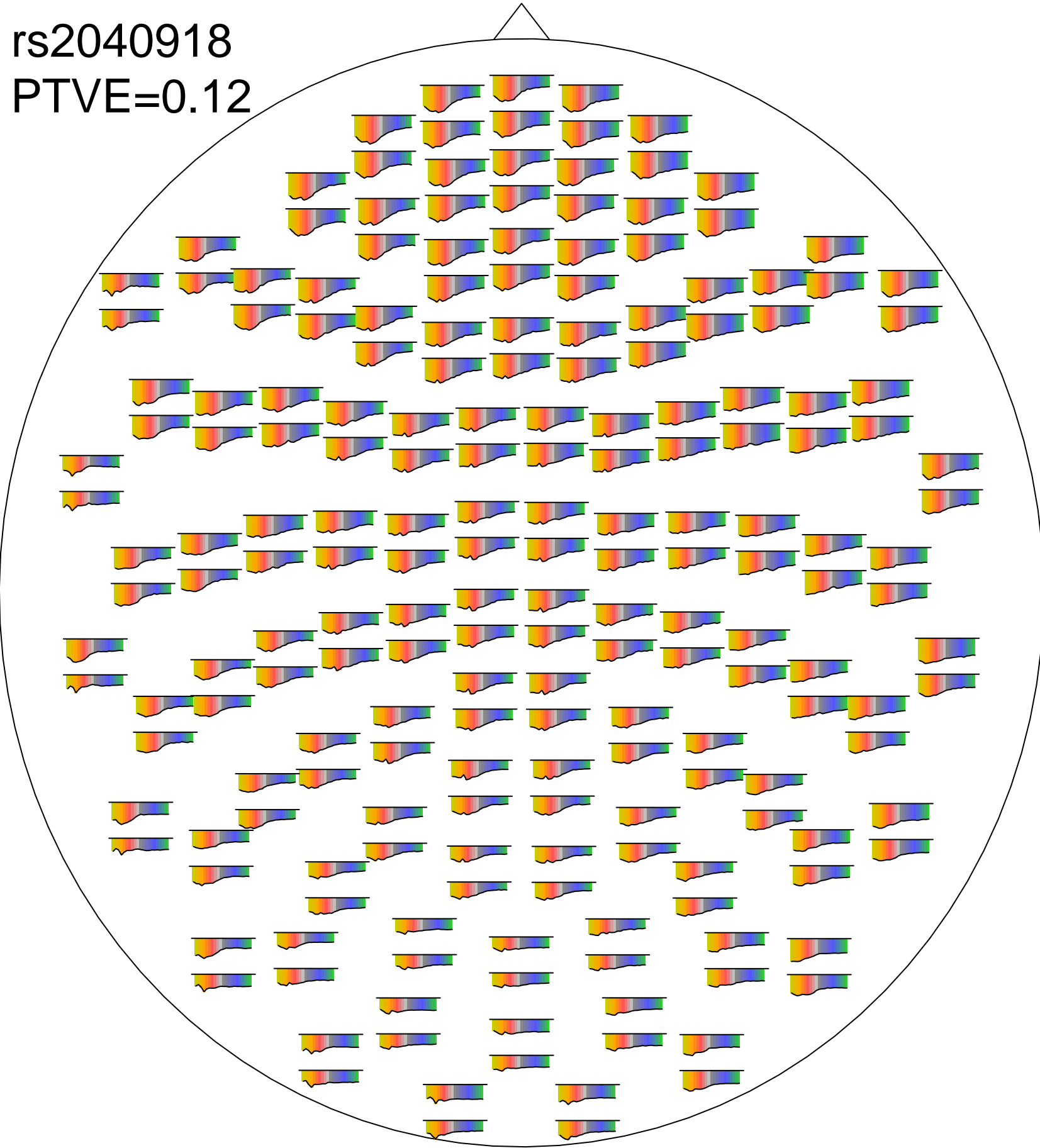

rs6454976  
PTVE=0.062

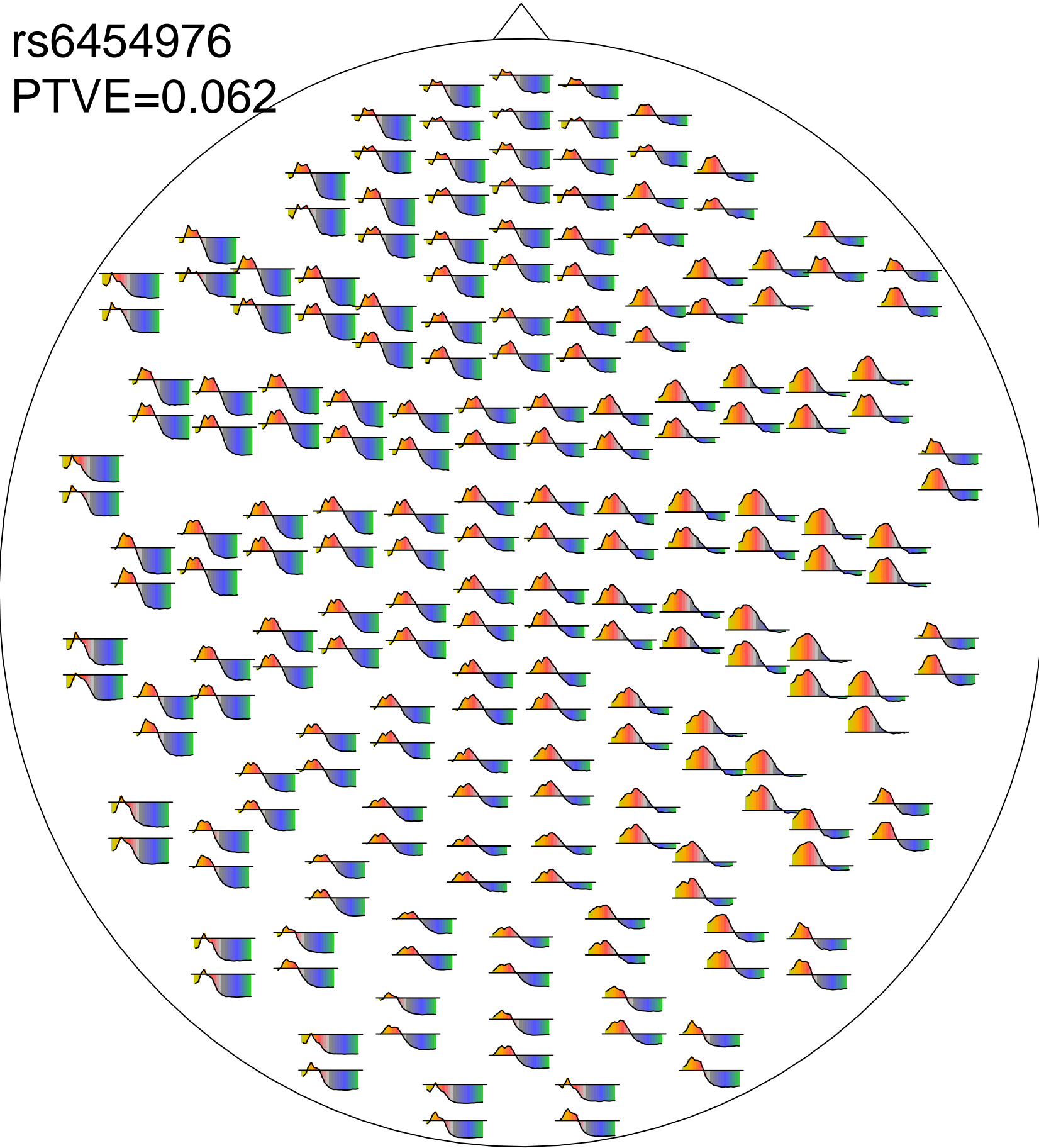

rs13057362  
PTVE=0.054

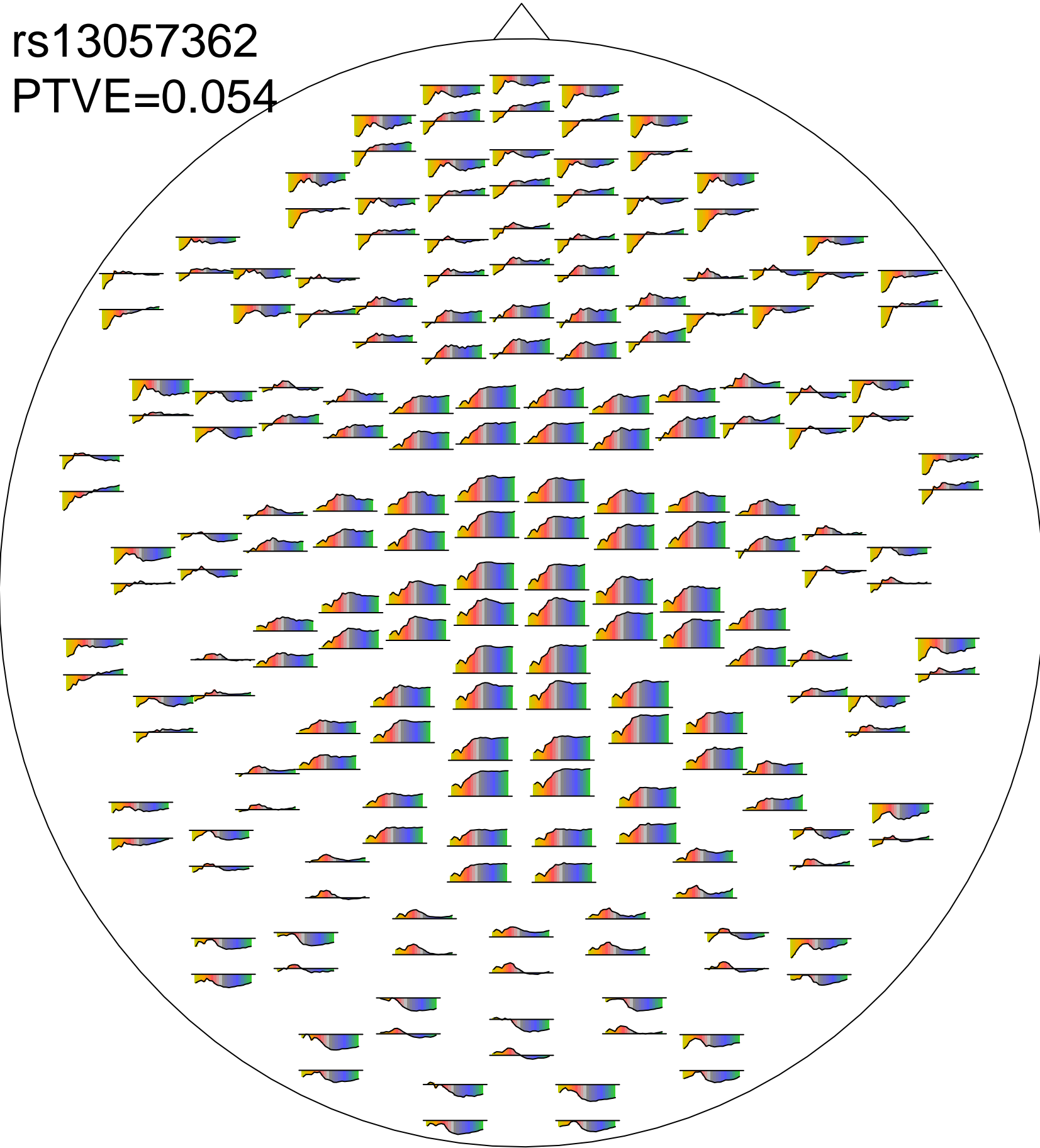

rs2241220  
PTVE=0.053

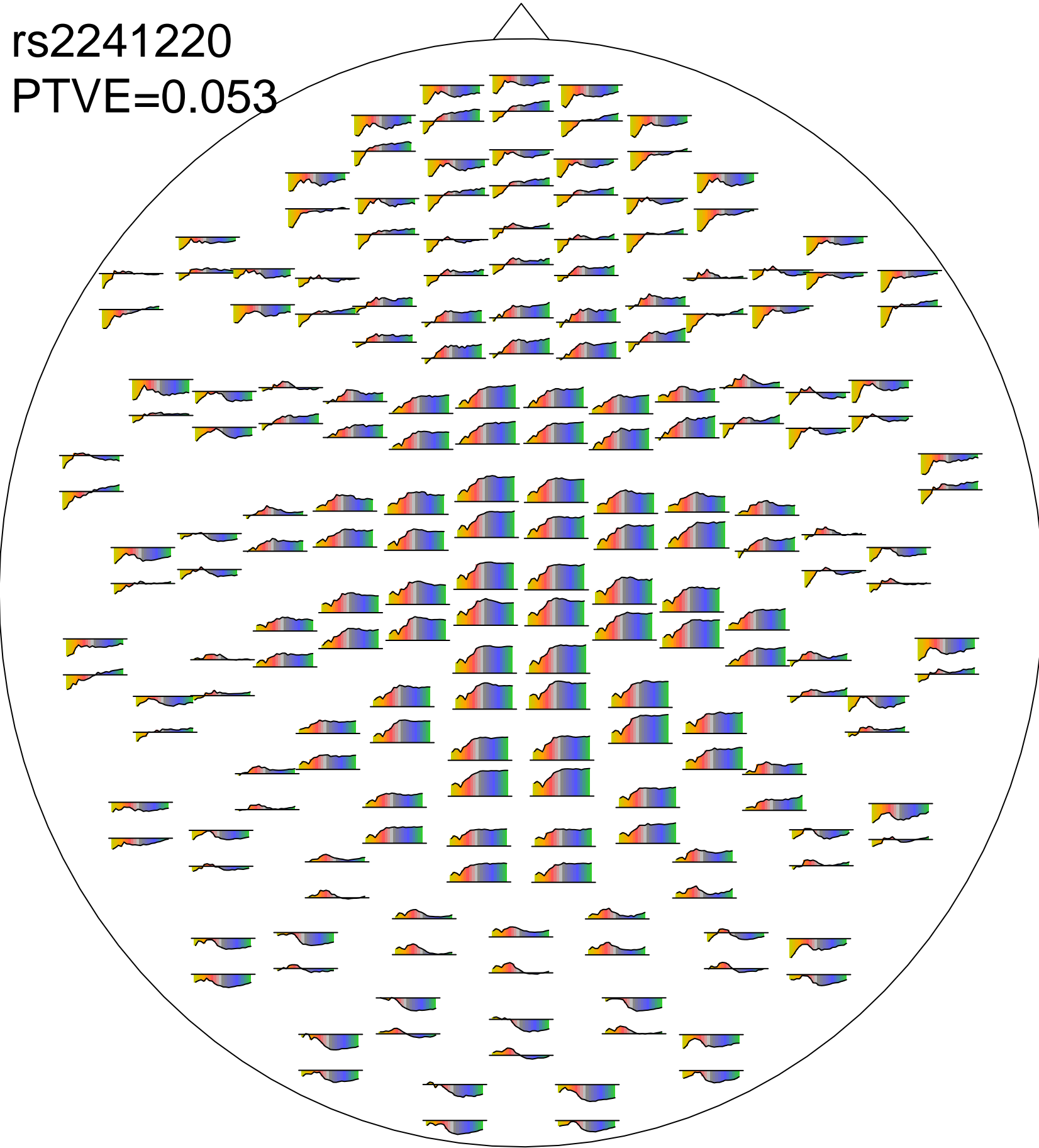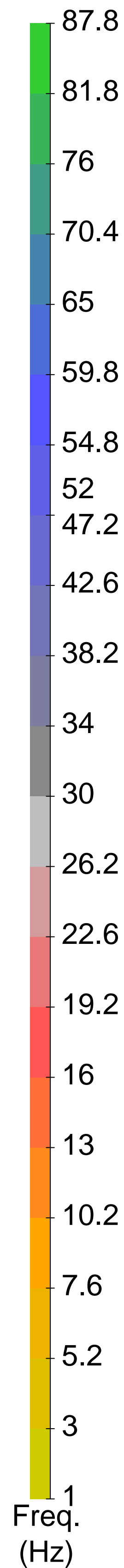

Supplement: Supplementary file 1 — Supporting Information S1 [file HBM-40-1391-s001.pdf]

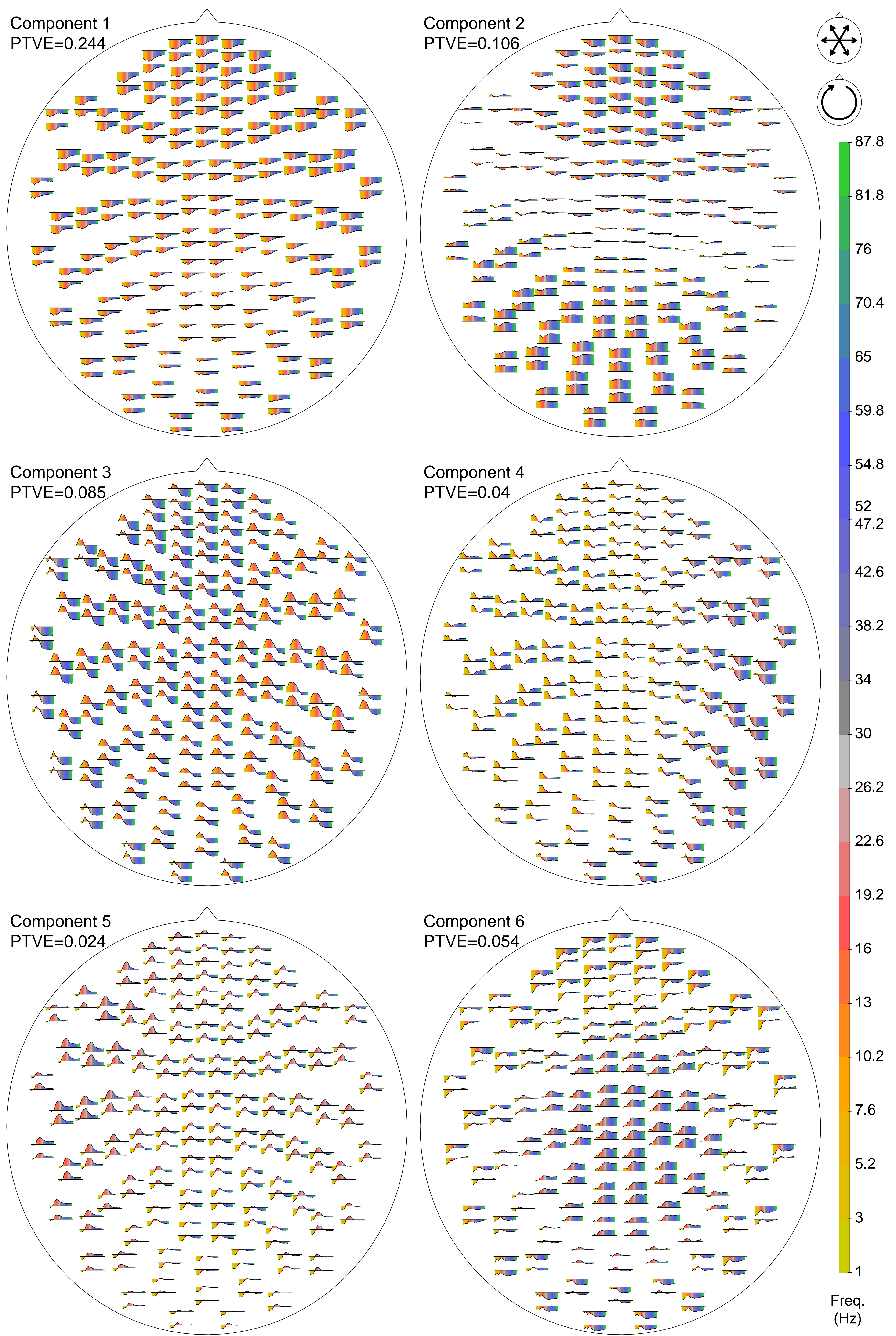

Supplement: Supplementary file 2 — Supporting Information S2 [file HBM-40-1391-s002.pdf]

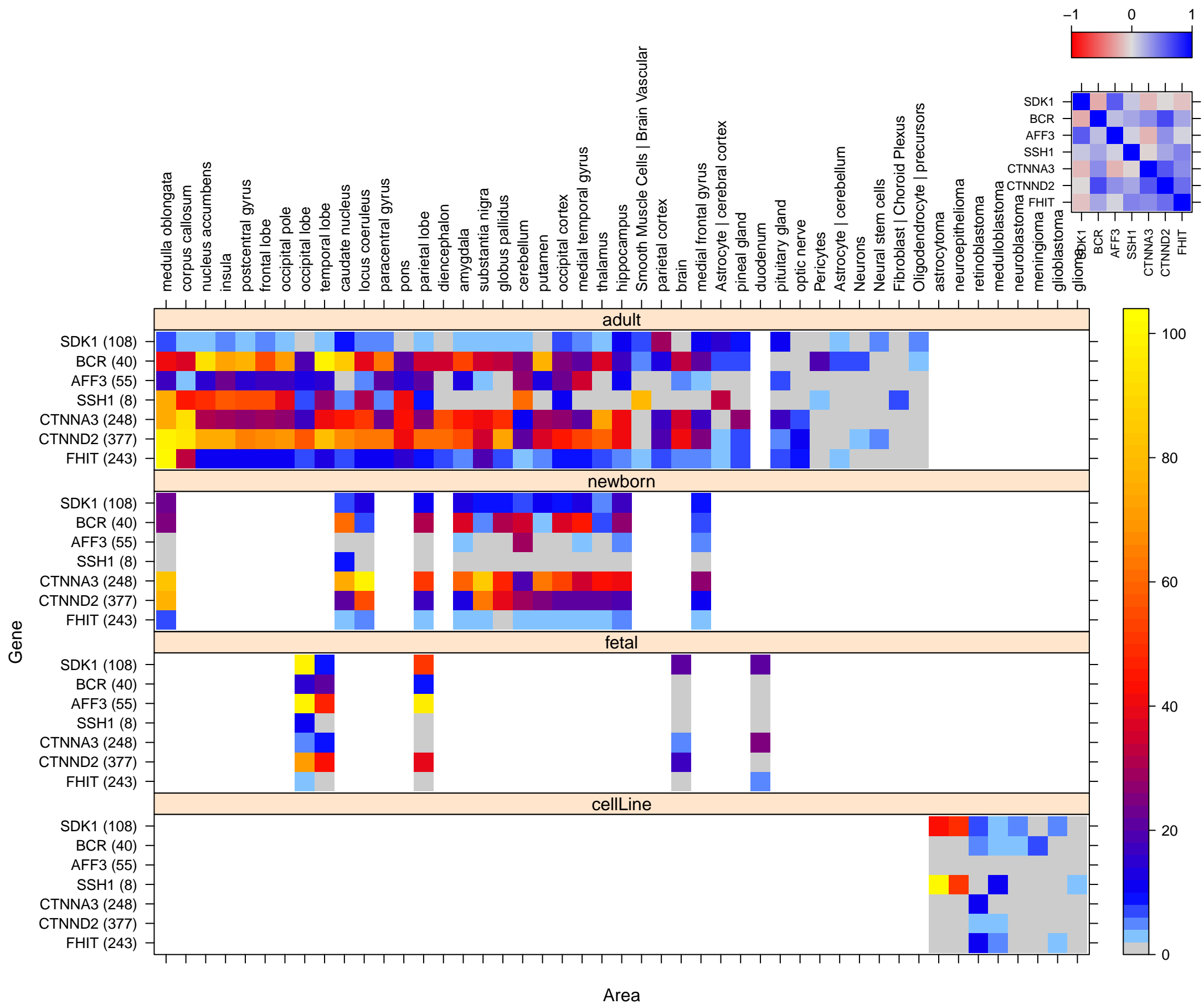

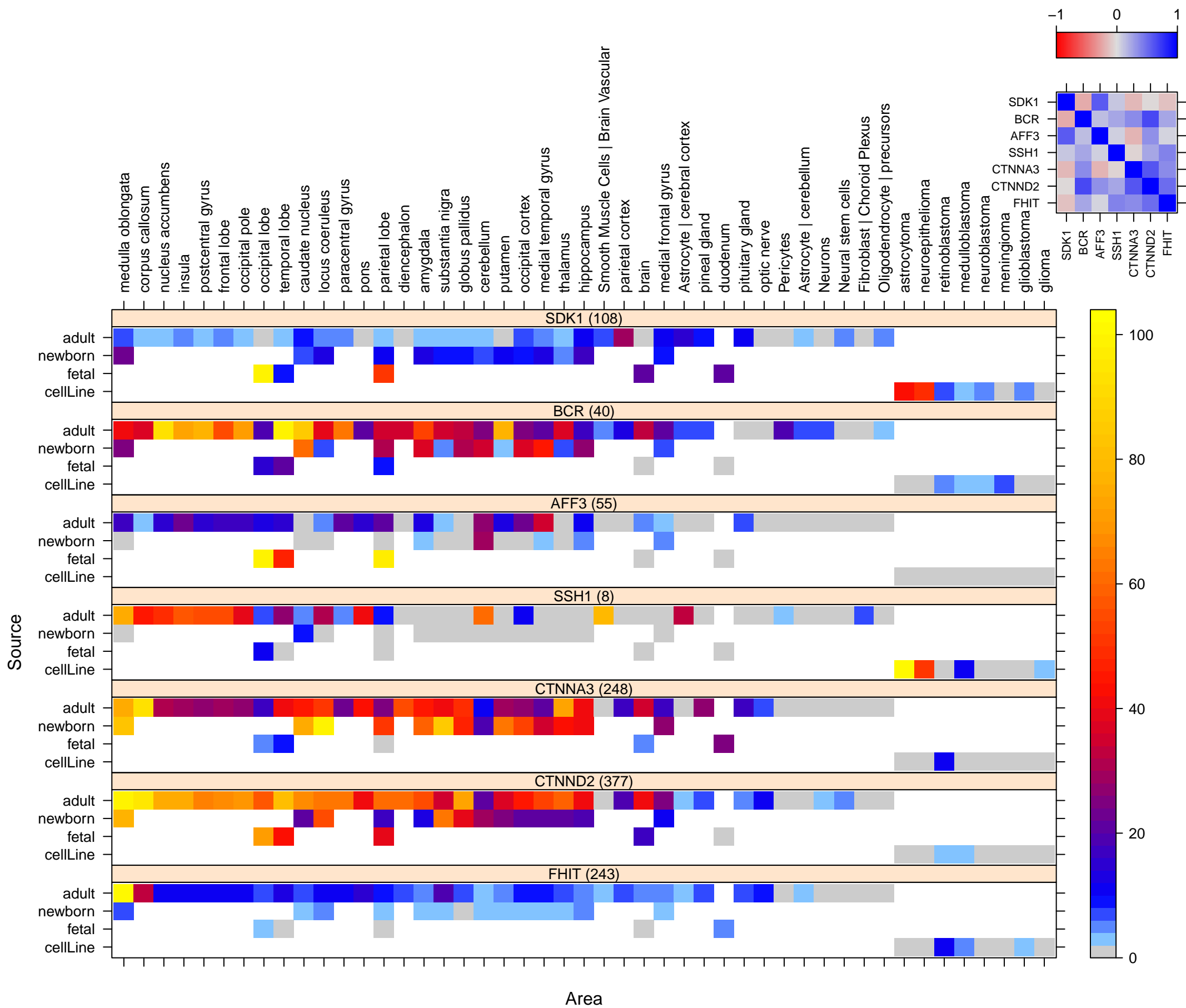

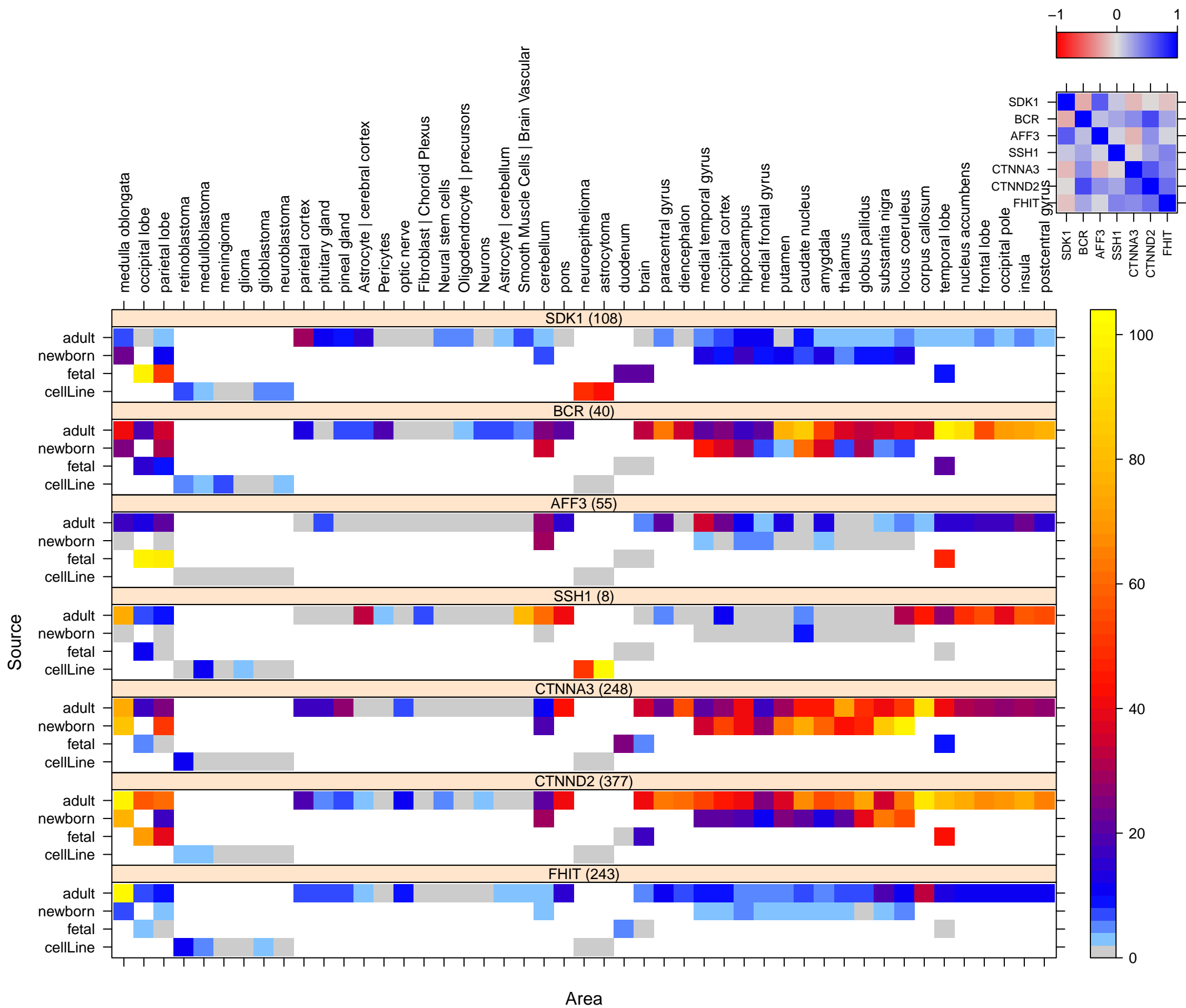

Supplement: Supplementary file 3 — Supporting Information S3 [file HBM-40-1391-s003.pdf]
