# Supplementary material for: Discovering heritable modes of MEG spectral power
Source: Hum Brain Mapp. 2019 Jan 1;40(5):1391–402. doi: 10.1002/hbm.24454 (PMC6590382; doi:10.1002/hbm.24454)
Supplement: Supplementary file 4 — Supporting Information S4 [file HBM-40-1391-s004.pdf]

Supplementary material for “Discovering modes of MEG spectral power with genetic associations”

Eemeli Leppäaho Hanna Renvall Elina Salmela Juha Kere
Riitta Salmelin Samuel Kaski

January 1st, 2019

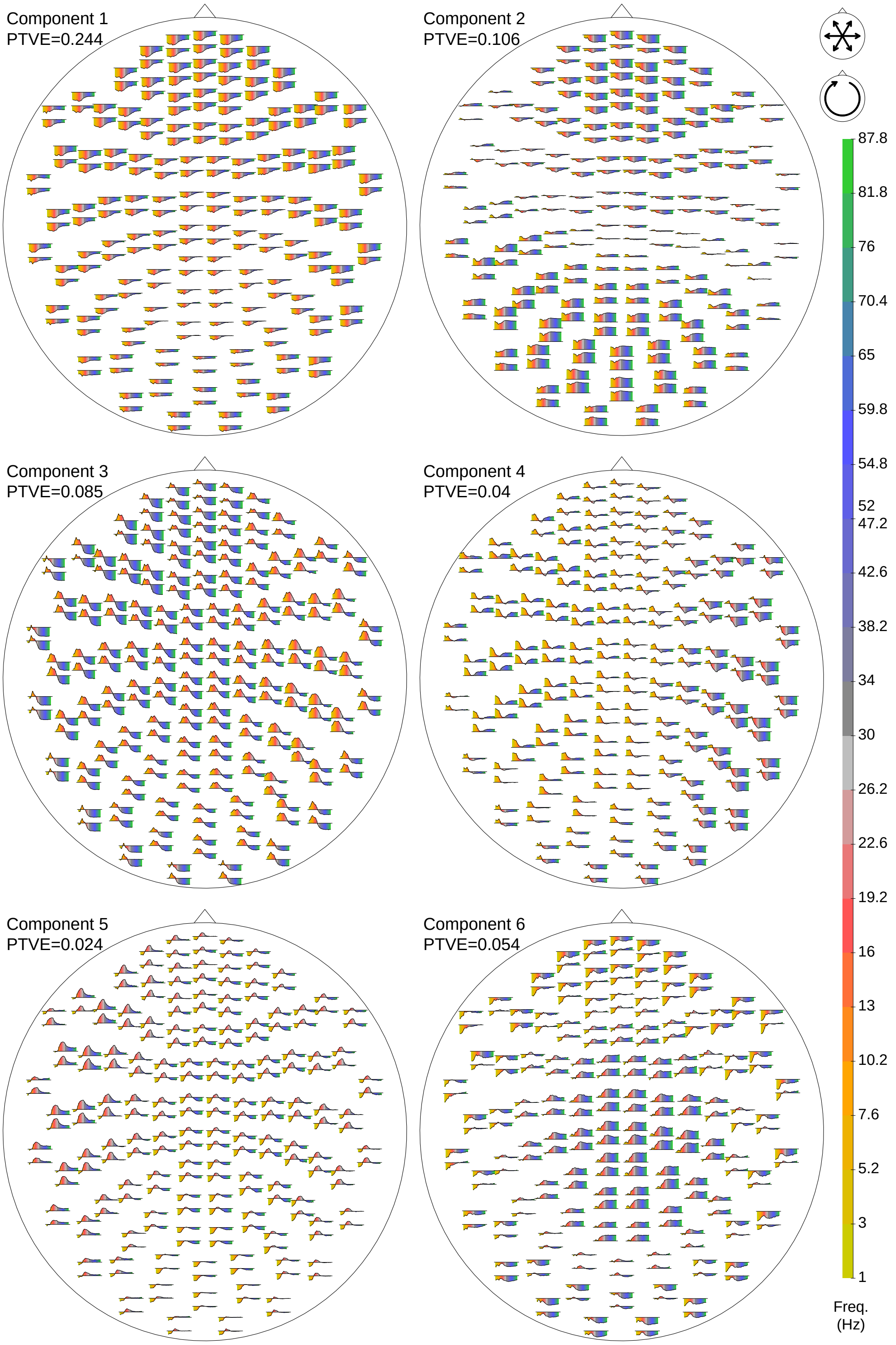

Figure 1: Extension of Figure 3 showing all the gradiometers. Six BRRR components best explaining the brain activity differences between families. The varying component weights over frequencies (1-88 Hz) on each gradiometer sensor are illustrated on the MEG sensor plane. For visualization, each frequency bandwidth (in Hz) is given a color shown on the right. The measurement helmet is viewed from above, flattened onto a plane, with the nose pointing upward. The 204 planar gradiometers of the Vectorview system are arranged in 102 locations along the helmet. At each location, there are two orthogonally oriented sensors, with the top and bottom curve depicting the output of the gradiometer most sensitive to longitudinally and latitudinally oriented currents (schematic heads in the top right corner), respectively.

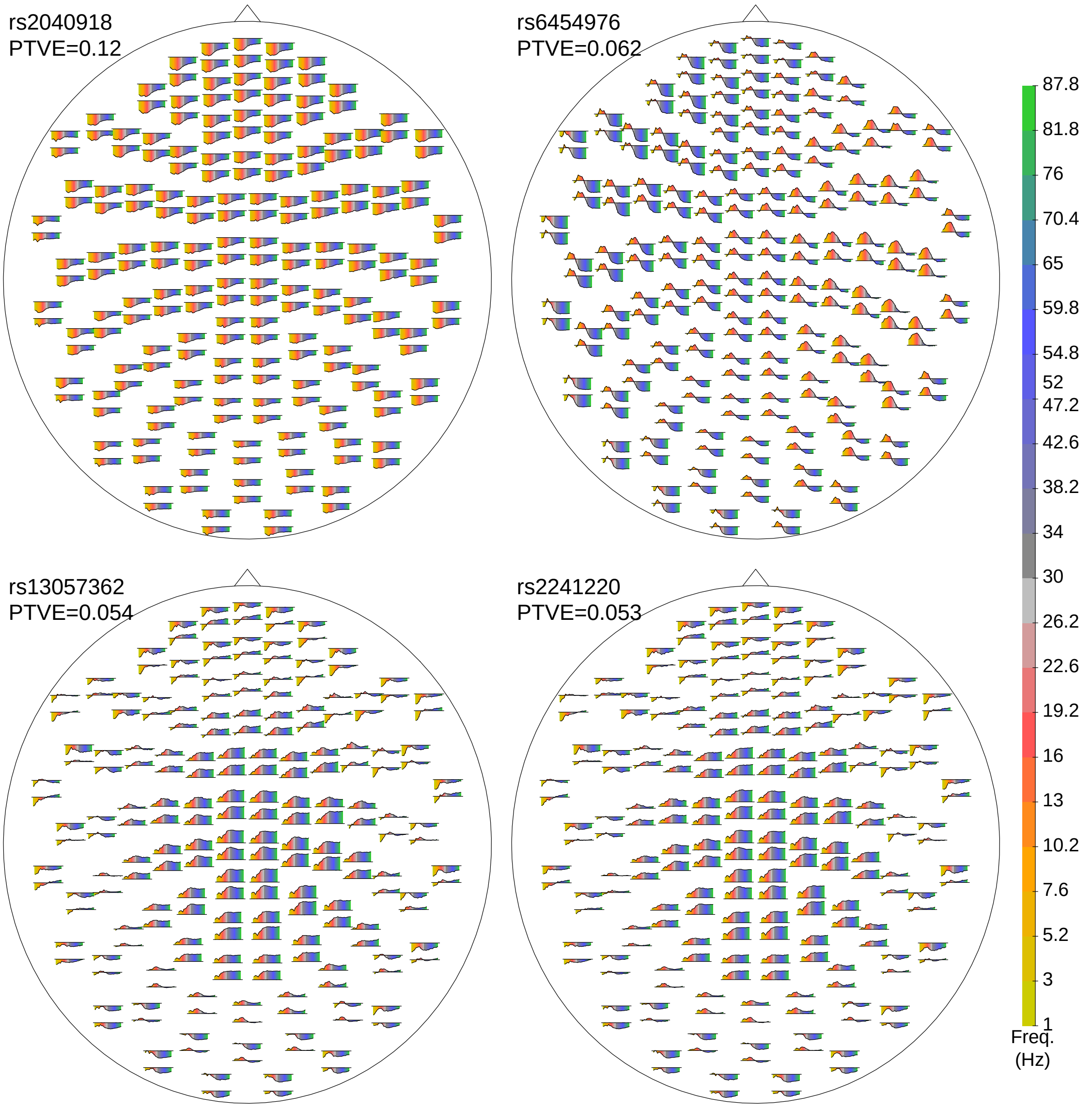

Figure 2: Extension of Figure 5 showing all the gradiometers. Components significantly explained by individual SNPs, with proportion of total variance explained (PTVE) shown. Each associated SNP explained strongly the total spectral power, similar to rs2040918 (top left) here; SNPs strongly explaining other structure as well are illustrated. The varying component weights over frequencies (1-88 Hz) on each gradiometer pair are illustrated on the MEG sensor plane. Each frequency bandwidth (in Hz) is given a color shown on the right.

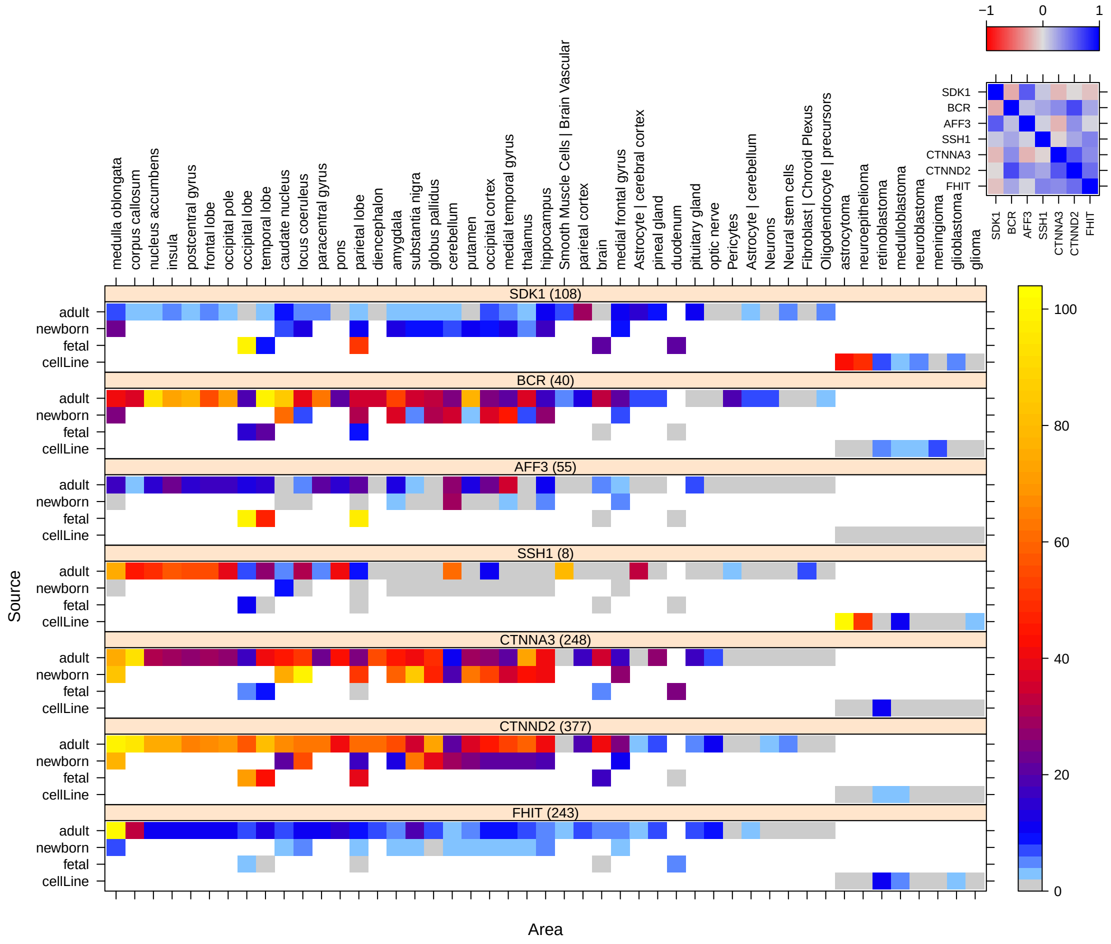

Figure 3: Gene expression profiles of the associated genes in cells with origin in different brain areas. Expression values below 2 are shown as grey, and values above it are scaled for each gene to range from 2 to 100; the actual maximal values for each gene are in parentheses. Genes FRMD1 and CES5AP1 are not shown, as they had no expression in the brain. Top right: correlations between the expression levels of the associated genes in the brain (blue: positive, red: negative).
